# WHO Better Outcomes in Labour Difficulty (BOLD) project: innovating to improve quality of care around the time of childbirth

**DOI:** 10.1186/s12978-015-0027-6

**Published:** 2015-05-26

**Authors:** Olufemi T Oladapo, João Paulo Souza, Meghan A Bohren, Özge Tunçalp, Joshua P Vogel, Bukola Fawole, Kidza Mugerwa, A Metin Gülmezoglu

**Affiliations:** Department of Reproductive Health and Research including UNDP/UNFPA/UNICEF/WHO/World Bank Special Programme of Research, Development and Research Training in Human Reproduction (HRP), World Health Organization, Avenue Appia 20, Geneva, 1201 Switzerland; Department of Social Medicine, Ribeirao Preto School of Medicine, University of São Paulo, Ribeirao Preto, São Paulo, 14010 Brazil; Department of Population, Family and Reproductive Health, Johns Hopkins Bloomberg School of Public Health, 615 N. Wolfe Street, Baltimore, MD 21205 USA; Department of Obstetrics and Gynaecology, College of Medicine, University of Ibadan, Ibadan, Nigeria; Department of Obstetrics and Gynaecology, Makerere University, Kampala, Uganda

**Keywords:** Quality of care, Partograph, Labour dystocia, Clinical decision-support, Service design, Childbirth

## Abstract

As most pregnancy-related deaths and morbidities are clustered around the time of childbirth, quality of care during this period is critical to the survival of pregnant women and their babies. Despite the wide acceptance of partograph as the central tool to optimize labour outcomes for over 40 years, its use has not successfully improved outcomes in many settings for several reasons. There are also increasing questions about the validity and applicability of its central feature – “the alert line” – to all women regardless of their labour characteristics. Apart from the known deficiencies in labour care, attempts to improve quality of care in low resource settings have also failed to address and integrate women’s birth experience into quality improvement processes. It was against this background that the World Health Organization (WHO) embarked on the Better Outcomes in Labour Difficulty (BOLD) project to improve the quality of intrapartum care in low- and middle-income countries. The main goal of the BOLD project is to reduce intrapartum-related stillbirths, maternal and newborn mortalities and morbidities by addressing the critical barriers to the process of good quality intrapartum care and enhancing the connection between health systems and communities. The project seeks to achieve this goal by (1) developing an evidence-based, easy to use, labour monitoring-to-action decision-support tool (currently termed **S**implified, **E**ffective, **L**abour **M**onitoring-to-**A**ction – SELMA); and (2) by developing innovative service prototypes/tools, co-designed with users of health services (women, their families and communities) and health providers, to promote access to respectful, dignified and emotionally supportive care for pregnant women and their companions at the time of birth (“Passport to Safer Birth”). This two-pronged approach is expected to positively impact on important domains of quality of care relating to both provision and experience of care. In this paper, we briefly describe the rationale for innovative thinking in relation to improving quality of care around the time of childbirth and introduce WHO current plans to improve care through research, design and implementation of innovative tools and services in the post-2015 era.

Please see related articles ‘http://dx.doi.org/10.1186/s12978-015-0029-4’ and ‘http://dx.doi.org/10.1186/s12978-015-0028-5’.

## Background

Global efforts to meet Millennium Development Goals (MDG) 4 and 5 have led to remarkable progress towards reducing preventable maternal and newborn mortality. However, reflections during the last MDG years indicate that a lot still needs to be done to address the unfinished agenda of ending preventable maternal and newborn deaths, particularly in high burden countries [1]. As increasing number of births take place in health facilities, poor quality of care in those settings have become more prominent as a barrier to reducing preventable deaths. With 44% of stillbirths, 73% of newborn deaths and 61% of maternal deaths occurring around the time of childbirth and in the first postpartum week, quality care during this period is critical to the survival of pregnant women and their babies [2]. This realisation has led to a global shift towards investment in quality of care during labour and childbirth as the most impactful and cost effective strategy to save millions of lives by 2025 [3]. While individual evidence-based interventions to avert and reduce maternal, fetal and neonatal mortality and severe morbidity are well known, there is limited evidence on how they can be effectively implemented. It is widely agreed that identification and appropriate management of women at high risk of labour complications, careful supervision of labour and childbirth, prompt use of effective interventions and essential newborn care would avert the majority of intrapartum-related maternal and perinatal deaths [3]. Yet, key evidence gaps remain on how best to effectively integrate and apply these measures in low- and middle income countries. This paper briefly describes the rationale for innovative thinking in relation to improving quality of care around the time of childbirth and introduces WHO current plans to improve care through research, design and implementation of innovative tools and services in the post-2015 era.

### Why innovation is needed

For over 40 years, the partograph has been the central tool for risk identification and intervention during the course of labour, and it is universally recommended for labour management. Despite its wide acceptance and implementation globally, the use of the partograph has not successfully improved birth outcomes in many settings due to several factors. Notable among these factors are incorrect or inconsistent use, time constraints, shortage of skilled workforce and lack of knowledge of the partograph [4-6]. Simultaneous monitoring of women in labour and deriving timely and appropriate actions is particularly challenging for health workers in labour units with staffing and equipment shortages, especially for those with non-specialist training. Furthermore, there is no clear evidence that the use of partograph has a positive impact on important clinical outcomes [7]. Additionally, there is increasing evidence that the pattern of spontaneous labour progression may differ considerably from Friedman’s reports (the 1 cm/hour rule) which informed the foundation of the partograph [8-10]. While there is general agreement that the partograph use may not be clinically effective in reducing adverse health outcomes, there is currently no other alternative to partograph for labour monitoring. Recent innovations in this area have focused on different presentations of partograph, such as Partopen and electronic partograph, without challenging its clinical foundations [11,12]. Overcoming the challenges of the currently available labour tools requires innovative thinking that revisits the basis of the partograph, with the aim of developing a tool that provides customized, evidence-based guidance on labour monitoring and actions, and yet is easy to use and interpret. The need for innovation in this area has become even more crucial, as obstetric practices have evolved since the partograph was first developed, given the declining use of instrumental vaginal delivery and high rates of unnecessary labour augmentation and caesarean section [13,14]. Therefore, advancement in labour monitoring and appropriate decision-making in a way that preserves conservative labour management is justified.

In technical terms, the partograph is a bi-dimensional classifier^a^ with the intended attribute of stratifying women into high or low risk of adverse outcomes during the course of labour. The partograph uses its central feature – “the alert line” – to separate women into those who are likely to experience prolonged labour (and its possible consequences) in the absence of any intervention, from those with normal labour progress who do not require any particular intervention. This discrimination is dependent on two parameters and assumes that cervical dilatation is a constant function of time for every woman. This classification disregards other maternal characteristics that could influence cervical dilatation as labour progresses, which may not be captured or considered in routine labour monitoring. As an example, the inclusion of another variable such as the woman’s parity into the equation alters this bi-dimensional classification as the progress and outcomes of labour tend to vary with parity. Recent technological advances may facilitate incorporating multiple variables and help to chart a natural course of labour progression for individual woman. Artificial intelligence (AI) techniques now offer the ability to use non-linear data relationships to develop multi-dimensional classifiers that could be applied to predict the expected progress and labour outcomes of a woman. Research has shown that AI techniques (such as neural networks, fuzzy logic, support vector machines and dynamic mathematical modelling) can aid in the diagnosis of disease states, assessment of treatment outcomes, and appropriate timing of interventions and have been successfully applied in cardiology, ophthalmology and neurology [15]. Yet, there is presently no AI tool for simplifying labour management in any setting.

Apart from issues related to care provision during labour, novel approaches are also needed to improve the experience of care by pregnant women and their families during childbirth. Overmedicalization of childbirth has significantly undermined women’s experience at birth and is partly responsible for poor use of skilled care in low-income countries [16]. However, the provision of high quality care during labour and childbirth requires the integration of important elements that address not just the provision but also experience of care.

While evidence based practices that could be applied to effectively improve care provision are well known, the non-clinical interventions to improve birth experiences are often not well understood, contextual and may not be easily generalizable. As in the non-health service delivery organizations, health systems, particularly in low-income countries, must begin to operationalise the concept of medical staff as ‘service providers’ and pregnant women as ‘clients’ and strive to continuously innovate and improve health services in ways that are more effective, user-friendly and desirable for their clients. Unlike in other service delivery organizations, quality improvement process in maternal health in low-resource settings often lacks consumer feedback because health system designs are largely based on the perspectives of health providers and managers. Over the last decade, there is increasing recognition that the inclusion of the perspectives of those who access care is crucial to the quality improvement process and health systems in high-income countries have used this approach to improve experience of care and ultimately health outcomes [17,18]. Until now, the integration of values and preferences of women and their families into health service improvement in low- and middle-income countries largely remains unexplored.

### The WHO Better Outcomes in Labour Difficulty (BOLD) project

Based on the above reflections, the World Health Organization (WHO) embarked on the BOLD project to improve the quality of intrapartum care in under-resourced settings. This project aligns with the new WHO vision of a world where “*every pregnant woman and newborn receives quality care throughout pregnancy, childbirth and the postnatal period*” and its underlying framework for quality of care [19].

The main goal of the BOLD project is to address critical barriers to the process of good quality intrapartum care and enhance the relationships between health systems and communities. Through this approach, the project aims to reduce intrapartum-related stillbirths, maternal and newborn mortalities and morbidities. WHO seeks to achieve this goal by (1) developing an evidence-based, easy to use, labour algorithm that could serve as the backbone for a user-friendly AI labour monitoring-to-action tool (currently termed **S**implified, **E**ffective, **L**abour **M**onitoring-to-**A**ction – SELMA); and (2) by developing innovative service prototypes/tools, co-designed with users of health services (women, their families and communities) and health providers and managers, to promote access to respectful, dignified and emotionally supportive care for pregnant women and their companions at the time of birth (“Passport to Safer Birth”). This two-pronged approach is expected to impact on important domains of quality of care relating to both provision and experience of care.

**Figure 1:**
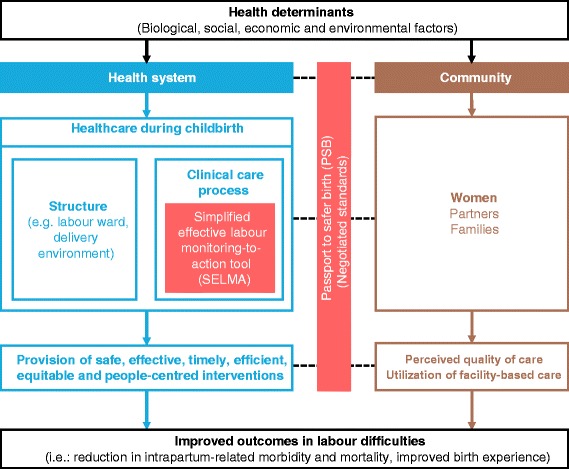
BOLD project conceptual framework.

The BOLD project is using an innovative approach to increase efficiency of the care process in the health system while stimulating the community to demand and use this improved care through research, design and implementation of innovative tools and services. Figure 1 presents the conceptual framework for the project, which connects SELMA and Passport to Safer Birth. The framework draws substantially from previous conceptual models on social determinants of health [20], quality of care [21], and skilled birth attendance [22]. It acknowledges the role of biological, social, economic and environmental determinants in shaping the health of the population, and the importance of the health system to influence health outcomes and promote equity. The framework highlights the entry points for SELMA and Passport to Safer Birth within this broader context, and how these tools could contribute to better process and outcomes for women and their babies.

Within this framework, we hypothesize that the quality of intrapartum care can be improved through the use of SELMA within facilities, by optimizing labour management and reducing unnecessary medical interventions and practices. Our assumption was that the development of a tool that optimizes task shifting, individualised childbirth care with a high potential for favourable outcome will increase providers’ skills and competence and motivate them to provide and respond to increasing demand for quality care. The ‘negotiated standards’ underpinning the Passport to Safer Birth will play a critical role in improving women’s birth experience at the facility, and thus their perception of quality of care [23]. A common agreement on what is scientific, feasible and user-centred between health system and community members will lead to service improvement and ultimately better birth outcomes. Improvement in the quality of care will in turn improve women’s satisfaction, perceived quality and birth experience, and thus stimulate demand for quality childbirth care by the entire community.

## Conclusion

The first challenge to achieving the goal of the BOLD project is to identify appropriate research settings to gather high quality data that will be used to develop the integral tools. However, the WHO’s multicountry survey network provides an adequate pool that will enable selection of hospitals with appropriate standard of childbirth care and community linkage. The development of SELMA will be based on a cohort study of women giving birth in health facilities in Nigeria and Uganda. Passport to Safer Birth will be the product of combined qualitative and health service design research techniques to be conducted in the same countries. The methodological details of the research work for the development of both tools are well described in their protocols that are published within this series [23,24]. We envision that, following the above activities, further research will be conducted to evaluate the effectiveness and implementation of SELMA and Passport to Safer Birth. Our findings and products will ultimately be channelled towards improvements of quality of labour and childbirth care and reduction in adverse maternal and newborn outcomes in low- and middle-income countries.

## Endnotes

^a^Classifiers are tools designed to identify the group to which a new observation belongs.

## Abbreviations

AI: Artificial intelligence
BOLD: Better Outcomes in Labour Difficulty
MDG: Millennium Development Goals
SELMA: Simplified, Effective, Labour Monitoring-to-Action
WHO: World Health Organization
